# Ambulatory Assessment Facilitates Longitudinal Assessment of the Mental Health Impact of COVID-19 in Older Adults

**DOI:** 10.1093/geroni/igaf122.1891

**Published:** 2025-12-31

**Authors:** Cuiling Wang, Chenxin Zhang, Qi Gao, Angel Garcia De La Garza, Richard Lipton, Carol Derby, Mindy Katz

**Affiliations:** Albert Einstein College of Medicine, Bronx, New York, United States; Albert Einstein College of Medicine, Bronx, New York, United States; Albert Einstein College of Medicine, Bronx, New York, United States; Albert Einstein College of Medicine, Bronx, New York, United States; Albert Einstein College of Medicine, Bronx, New York, United States; Albert Einstein College of Medicine, Bronx, New York, United States; Albert Einstein College of Medicine, Bronx, New York, United States

## Abstract

The impact of the COVID-19 pandemic on the mental health of community-dwelling older adults from diverse racial and ethnic backgrounds is not well known, partly due to a lack of longitudinal studies with systematic and continuous assessments before, during and after the pandemic. In the Einstein Aging Study (EAS), both conventional and ambulatory digital assessments of various clinical and behavioral measures, including mental health, were administered annually since 2017. The ambulatory assessment consisted of a 2-week burst of digital survey and cognitive measures obtained 6 times per day. Using ambulatory assessment of mood, stress, anxiety, loneliness, depression, we evaluated changes in mental health through stages of the COVID-19 pandemic while taking account of age-related changes. The impact of age, sex and race/ethnicity, as well as various factors including social economic status (SES), social network, personality, were also examined. Multi-level mixed effects models for the intensive longitudinal mental health outcomes were applied to examine simultaneously the trajectories during follow-up time and changes over the calendar time based on phases of the COVID-19 pandemic. Among n = 320 EAS participants, we observed resilience in mental health as shown by quick recovery after an initial worsening at the beginning of the COVID-19 pandemic as indicted by the calendar time factor, and there was an overall decline trend during follow-up time. The role of demographics, SES, social network, personality, and behaviors during COVID-19 pandemics are discussed.

